# Positional Homology in Bacterial Genomes

**Published:** 2007-01-14

**Authors:** Ingrid J. Burgetz, Salimah Shariff, Andy Pang, Elisabeth R. M. Tillier

**Affiliations:** 1,2,3 Ontario Cancer Institute, University Health Network, Toronto, Ontario, Canada; 3 Dept. of Medical Biophysics, University of Toronto and

**Keywords:** Bacterial genomes, orthology, paralogy, gene order

## Abstract

In comparative genomic studies, syntenic groups of homologous sequence in the same order have been used as supplementary information that can be used in helping to determine the orthology of the compared sequences. The assumption is that ortholo-gous gene copies are more likely to share the same genome positions and share the same gene neighbors. In this study we have defined positional homologs as those that also have homologous neighboring genes and we investigated the usefulness of this distinction for bacterial comparative genomics. We considered the identification of positionaly homologous gene pairs in bacterial genomes using protein and DNA sequence level alignments and found that the positional homologs had on average relatively lower rates of substitution at the DNA level (synonymous substitutions) than duplicate homologs in different genomic locations, regardless of the level of protein sequence divergence (measured with non-synonymous substitution rate). Since gene order conservation can indicate accuracy of orthology assignments, we also considered the effect of imposing certain alignment quality requirements on the sensitivity and specificity of identification of protein pairs by BLAST and FASTA when neighboring information is not available and in comparisons where gene order is not conserved. We found that the addition of a stringency filter based on the second best hits was an efficient way to remove dubious ortholog identifications in BLAST and FASTA analyses. Gene order conservation and DNA sequence homology are useful to consider in comparative genomic studies as they may indicate different orthology assignments than protein sequence homology alone.

## Introduction

Homologous sequences have been further defined as paralogs when they are the result of a duplication event, or as orthologs when they are the result of a speciation event (Fitch, 1970, Fitch 2000, Koonin 2005). The definition of orthology therefore does not allow us to precisely follow the history of a particular DNA sequence, particularly when multiple duplication and speciation events have obscured the historical relationship between homologs.

**Figure 1:**
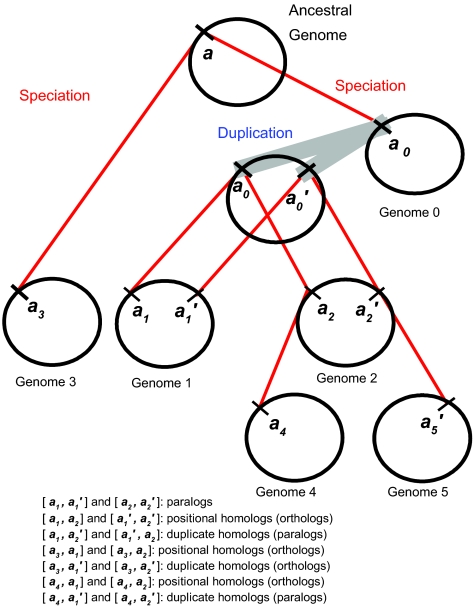
Defining positional and duplicate homology A gene ***a*** in an ancestral genome at the root of the tree follows the phylogeny of its host genome with speciation (thin lines). In Genome 0 the homologous gene **a0** is duplicated (thick lines) yielding a paralog ***a***′***0*** into another genomic position. Homologs of these two genomes are then found in genomes 1 and 2 after speciation. Genome 4, a descendant of genome 2 loses one of the gene’s copies while genome 5 has lost the other copy. Genome 3 also has a single copy of the gene that arose from speciation of the ancestor at the root, before duplication of the gene. Positional homologs are defined as homologs in the same ancestral genomic position. Duplicate homologs are defined when the homologs are different genomic positions and with the additional requirement that a positional homolog exists for at least one of the two genes.

An illustration of how genomic position can supplement sequence information is given in Figure 1. Duplication of gene *a_0_* in the genome 0 will result in paralog pairs [***a1,a***′***1***^]^ and [***a2,a***′***2***] after speciation into genomes 1 and 2. Pairs [***a1,a2***] are orthologs, whereas [***a1,a***′***2***] are paralogs because of the additional duplication step separating them. It is very difficult to distinguish these possibilities when only sequence homology between pairs of genomes is considered since the copies are, at first, identical. Other situations as depicted in the figure 1 add to the complexity, since speciation may have occurred prior to the duplication event (genome 3): the ho-molog in this genome has two orthologs in genomes 1 and genomes 2 which are not distinguished. If a genome loses one of the homologs subsequent to the duplication event, as occurs in genomes 4 and 5, then sequence homology based analyses may misidentify the orthology or paralogy relationships in comparisons of the remaining genes with those in other genomes. In order to help the analyses, we can obtain additional information by considering the genome context. In all the depicted scenarios in figure 1, when multiple homologs exist for a gene, then positional homologs always indicate orthologs while duplicate homologs in different genomic positions, possibly (but not always) indicate paralogs. Accurately determining positional homology can then greatly help in assigning orthology. In the Cannon and Young (2003) and Bourque, Pevzner and Tessler (2004) methods, the homologous sequences considered need not be annotated genes but simply homologous syntenic segments. In Kellis *et al.* (2003) , homologous predicted ORFs were used.

In a duplication event, even if an identical DNA sequence copy is made, it cannot occupy the exact same genomic position. It will therefore not be identical in its absolute position with respect to a genome marker such as the origin of replication, or in its relative position with respect to neighboring genes. Only in cases where a gene is tandemly or segmentaly duplicated, do copies conserve some aspect (absolute or relative) of the original position in the genome. In many cases, the copies can be distinguished from the original because of their new location.

For comparative genomic studies that are interested in gene order evolution, it is critical that the positional homologs be accurately identified in order to infer the numbers and types of genomic rearrangements that have occurred in the history of the genomes. Most algorithms for inferring phylogeny from gene order only permit a one-to-one orthologous relationship. When there are multiple orthologs, as in the case of lineage specific expansion, the positional homolog indicates which copy should be chosen to represent the gene for the algorithms to give the most parsimonious solution. When there is some conservation of the gene order between the genomes of two species, whether due to functional constraint or phylogenetic relatedness, the identification of homologs in the same position in both genomes can be taken as further evidence of their orthology. If more than one homolog to a gene is identified by an alignment algorithm, and if one of these is in the same genomic position, then that copy is the positional homolog and is most probably an ortholog. We define the other copies as *duplicate homologs.* The positional homologs are in the ancestral position and thus are *examplar* genes as defined by Sankoff (1999) in the context of the genome rearrangement problem.

For functional studies, the identification of homologs by sequence is often more important than their location, however it could be important to correctly identify the genomic context. This could be used to determine, for example, whether functional differentiation of the copies is attributable to a different genetic context (possibly gene regulation, or fusion to other domains) or/and the sequence differences between the copies.

Although gene order conservation rarely survives beyond the genus level, there is still considerable gene order conservation between many of the available sequenced bacterial genomes (Huynen and Bork 1998, Tamames 2001, Tillier and Collins 2000, Wong and Golding 2003; also see examples at http://www.uhnres.utoronto.ca/tillier/Xplots.html). The most enduring conservation of gene order is due to functional constraints, such as an operon. This is a property that has been exploited (Dandekar *et al*. 1998, Overbeek *et al*. 1999, Wolf *et al*. 2001, Snel, Bork and Huynen. 2002, Doerks *et al*. 2004) to identify functional interactions between genes (and with regulatory elements). However in many cases where closely related genomes are considered, conservation of gene order can simply be due to phylogenetic relatedness (Tillier and Collins 2000, Wong and Golding 2003). The consideration of gene order conservation for orthology determination is thus mostly useful in the comparison of closely related genomes and for conservation of ancestral function in the comparison of more diverged genomes.

Primarily homologous genes sharing the same position in the genome determine positional homology, and this position can be determined in several ways. Different methods will have different sources of errors in their estimate of ancestral homology. For example, we could consider genes within the same distance from some fixed point on a chromosome: a telomere or centromere, or in bacterial genomes, the origin of replication. However this approach will only be accurate if the reference point is indeed immovable. A more accurate approach would be to consider gene neighborhood (Zheng *et al*. 2005, Notebaart *et al*. 2005, for example), where positional homologs also have neighboring genes that are homologous. Variable numbers of homologous neighbors and variable distances from the query gene could be considered. This method can be erroneous in cases of tandem and segmental duplications because when syntenic segments are duplicated, genetic neighborhood becomes uninformative of positional homology. For this reason the approach would also not be generally useful for eukaryotic genomes particularly in the extreme case of whole genome duplication.

In this study we analyzed fully sequenced and annotated bacterial genomes and defined positional homologs as those that also shared at least one other homologous adjacent gene. We used sequence criteria (i.e. BLASTP) to identify within genome paralogs and between genome homologs and considered whether gene position was conserved. For the purpose of this study, tandemly repeated genes were ignored since they share the same position and cannot be distinguished via gene order. We also considered alignments at the DNA level in an attempt to confirm the positional homology of the genes. Subsequently, we determined the most efficient criteria for the selection of positional homologs when gene order is not known or not considered. Lastly we analyzed the COG database to identify positional homologs, and particularly ones that would not have been identified using only sequence information.

## Methods

### Test of positional homology based on gene neighbor conservation

**Figure 2:**
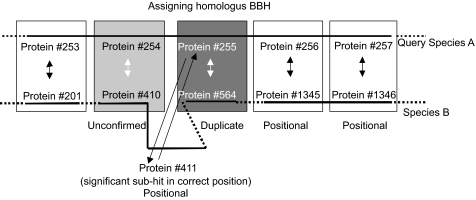
Confirming positional homology of BBH with gene order information In the comparison of genome 1 and genome 2, BBH are identified by BLASTP or FASTA. Bidirectional arrows indicate BBH, and single arrows indicate significant but not BBH hits. When the BBH of two neighbor proteins (i.e. #256 and #257) are also neighbor proteins (#1345 and #1346) then these are positional homologs and we call the BBH Confirmed. A BBH was considered Unconfirmed in the case where the BBH of neighbors (#254 and #255) are not neighbors (#410 and #564) and considered Duplicate if a sub-hit did include the neighbor protein (#411).

All sequenced bacterial genomes were obtained from NCBI genomes ftp server **NCBI bacterial ge-nome ftp site:** (ftp://ftp.ncbi.nih.gov/genbank/genomes/Bacteria/). Bi-directional best hits (BBH) were identified by BLASTP (Altschul *et al*., 1997) and FASTA analysis (Pearson, 1994) of the annotated protein products in all-by-all pairwise genome comparisons. Very lenient cut-offs were applied (E-value cut-off of 10 and a minimum alignment length of 30 amino acids). The BBH were then assessed in the following manner (shown graphically in Figure 2).

1. The BBH were considered confirmed positional homologs (*P*) if there was at least one neighboring pair of genes that were also BBH. To allow for gene fusion events (Enright *et al*. 1999, Marcotte *et al*. 1999), cases where the same gene was the best hit for two neighbor genes were included.
2. When a BBH could not be confirmed with neighbor genes, we considered the sub-hits of the query genes. If a subhit was to a gene with a neighbor BBH to the neighbor of the original query, then the original BBH was determined to be a duplicate homolog (*D*).
3. When neighbor genes did not confirm the BBH, and no sub-hits rendered them Duplicate, then these were considered Unconfirmed (*U*).
4. BBH confirmed with neighbors, but that also had subsequent hits with neighbor BBH confirmation were called positionaly Ambiguous (*A*).
5. The accuracy in identifying positional homologs is lowered in cases of segmental duplications (such that two or more neighboring genes have been duplicated). Duplications of neighboring gene pairs were first calculated by considering all hits within a single genome. A gene was considered part of a segmental duplication if it and a neighbor gene hit another pair of neighboring genes with E<0.01. When a gene was found to belong to a segmental duplication in a genome all its BBH with other genomes were then also considered Ambiguous (*A*). Table 1 lists the number of segmental duplications found in 13 of the bacterial genomes.

An estimate of the error in *P, D* and *U* was obtained by considering the neighbors of another randomly chosen protein in the genome.

### Estimates of substitution rates

Homologs were identified as BBH with BLASTP in all-by-all comparisons of 146 genomes. We further analyzed homolog pairs with E value less than 10^−10^ identified as duplicates using gene order information. The sequence of the query and its BBH were aligned using Smith-Watterman (Smith and Watter-man 1981) and the corresponding in-frame DNA alignments were obtained. Similarily, an alignment of the query sequence with the positional homolog was also obtained.

As a control, a set of alignments were obtained with E-values and percent identity of the BBH to the subsequent hit comparable to the first set of alignments, but in this case the BBH were positionaly confirmed as orthologous.

The Smith-Watterman alignments of BBH and subhits were further analyzed only if their alignment score ranking was in agreement with the BLAST analysis so as not to consider ambiguous cases where there is no assurance of the best and second best hits. PAML Codeml (Yang 1997, Yang and Neilson 2000) analysis was performed to calculate the rate of synonymous (dS) and non-synonymous (dN) substitutions and their ratio. The substitution rates were calculated using runmode = −2 (pairwise comparison). For rate comparisons, differences between rates were standardized by the sum of the standard errors in the estimated dS and dN in order to reduce the contribution of the most variable estimates. Alignments with dS=0 (no DNA or amino acid substitutions) were excluded from the analysis.

### Evaluating the efficiency of cut-offs for accurate positional homolog identification

E-value cut-offs, E-value spread (ratio of E-values from best and second best hits), and alignment length cut-offs were evaluated for accuracy in predicting the positional homology of the BBH. Those BBH identified as meeting the more restrictive cut-off criteria were then assessed as to whether they were confirmed positional homologs (*p*)**,** unconfirmed (*u*) or duplicates (*d*) as defined above. These numbers were compared to those obtained without using cut-offs as above. The positional homolog identification score (*I_s_*) was calculated for the comparison using:

(1) $$I_{s}=\frac{p}{P}-\frac{d}{D}$$

This score can be considered as a combined sensitivity and specificity scores. For *I_s_*, a perfect score is +1 (identified all positional, but no duplicate BBH) and a score of −1 indicates only identification of duplicate (not positional) homologs. All genome comparisons considered had *P* > 0, and in the rare cases where *D* = 0, the second term in Eq. 1 was ignored. Ambiguous identifications were also ignored in this analysis.

## Results

### Consideration of Neighbors

A sample of 123 pairwise genome comparisons that cover the whole range of sequence and gene order conservation was analyzed. For all comparisons, we determined by BLASTP the number of Bidirectional Best Hits that could be labeled positional homologs, unconfirmed, or duplicate with gene neighbor information as described in Methods and presented in Figure 2.

**Figure 3:**
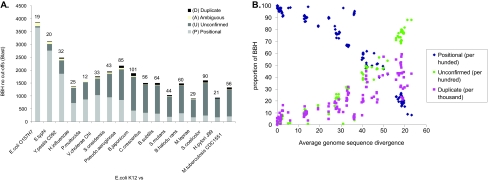
Positional homology of BBH in whole genome comparisons **3a** The number of BLAST BBH that are confirmed Positional homologs, Unconfirmed, Ambiguous and Duplicates with order information are given for several comparisons with the *E. coli* K12 genome. Genomes are given in order of increasing average sequence divergence (calculated as one minus the average sequence identity over all the BBH alignments). The number of duplicates is also shown above each bar in the graph. **3b.** The frequency of Positional and Duplicate holomologs (see legend) are plotted against the average divergence between all the BBH in 123 genome comparisons.

For comparisons of closely related genomes with strong gene order conservation, a large proportion of BBH were found to be positional homologs. The proportion of the BBH that were not positional homologs increased with the number of genome rearrangements and with sequence divergence. This can be seen in Figure 3a that shows comparisons to *E. coli* K12 and in Figure 3b, for all genome comparisons. Generally, the amount of positional homology decreases quickly with genetic distance (with the exception of the *Chlamydia* genomes which have more gene order conservation). Nevertheless, some gene order always remains due to the conservation of operons and gene clusters.

An interesting quantity is the frequency at which BBH were identified as duplicate homologs. These correspond to BBH for which an alternate subsequent hit was found in the same genomic location as the query. In this case, a positional homolog exists but the best BLAST hit did not identify the positional homolog but a duplicate copy (Figure 3). Their frequency (Figure 3b) was very low, particularly in close genome comparisons. This indicates that in most cases, the positional homolog is also the BBH, and that the protein sequence is more conserved with the positional ortholog than with the duplicate homologs. With increasing evolutionary distance, the number of BBH that were found to be duplicates increased, indicating more instances where the positional homologs has increased protein sequence divergence relative to other copies. The number of duplicates eventually decreased again as gene order is lost and we are unable to determine the positional homology of most genes.

The frequency of duplicates reported here is much lower than that found by Notebaart *et al*. (2005). In that study, the positional homolog was not the BBH in 29–38% of cases of duplicated genes, but the frequency was calculated only over recently duplicated genes, whereas we are considering the frequencies over all genes in a genome with a BBH in the other genome.

### DNA level comparisons

Homologs were identified using protein sequences in order to maximize the number of homologs found, but it is also possible to employ DNA level sequence analysis. In cases of paralog functional differentiation, protein sequence divergence may increase relative to DNA sequence divergence (Yang and Neilson 2000). We wanted to identify such cases where a BBH at the protein sequence level was found to be a duplicate homolog rather than a positional homolog, and recognize which copy was more diverged than at the DNA level. DNA sequences usually evolve much faster than protein sequences due to the possibility of synonymous substitutions, such that the lower substitution rate of the BBH should be also seen at the DNA level. The amount of sequence divergence of the homologs will depend on their particular history in terms of the relative timing of duplication and speciation events. However, we would expect under neutrality that the rates of DNA and protein divergence to be associated such that the rates of synonymous and non-synonymous substitutions are positively correlated in all cases outlined in Figure 1.

The analysis of the rate of evolution of both protein and DNA sequences may help to identify cases of functional differentiation of the positional homologs and duplicate homologs. The set of positional homologs that were not BBH were found using BLASTP analysis, aligned using Smith-Watterman and these alignments were then analyzed with Codeml from the package PAML (Yang 1997) in order to estimate rates of synonymous and non-synonymous substitutions. We also considered a control data set consisting of positionaly homologous BBH gene pairs with similar E-values and percent identities for the best hit and for the second best hit as in the set of duplicate BBH. In this set, the BBH was the positional homolog, but there was also a good subhit in another position. We expected lower levels of protein sequence divergence between the query gene and the BBH gene than with the sub-hit gene since the best hit should have a better alignment and better score. We indeed found this in the control data set (23276 query genes considered). As expected, positional homologs that were BBH showed significantly lower rates of both synonymous and of non-synonymous substitutions than the subhit pairs (p=2.5×10^−74^, in pairwise t-test for the difference in dS in the best hit and the subhit). Surprisingly this was not the case when the positional homologs were identified as a subhit pairs and not as the BBH (24524 query genes). In those cases, although the positional homologs showed a higher non-synonymous rate of substitution (p= 9.17×10^−31^) as expected, the synonymous rate of substitution was found to not be significantly higher than in the BBH pair (p = 0.4216). We found generally that subhits, when in the ancestral position, showed relatively *lower* levels of DNA sequence divergence, even though the rate of protein sequence divergence was *increased* relative to the BBH in a different genomic location.

Although that result is striking, the rates of substitution are very high in many of these comparisons and include comparisons between genomes with different base composition. Under these conditions, the estimates of dN and dS and of their standard errors by Codeml can be very unreliable (Yang and Neilsen 2000). It is unclear whether for a query in one genome, the comparison of the substitution rate estimates for the different hits in another genome, is meaningful in such cases of high dS values (Blanc, Hokamp, and Wolf 2003).

**Figure 4:**
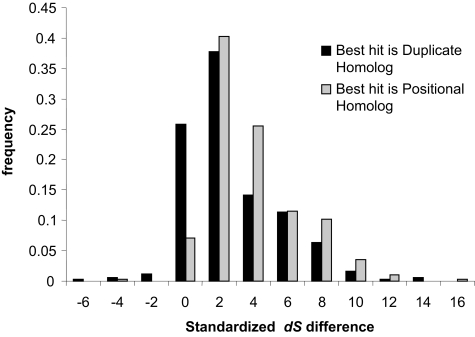
Standardized dS difference Frequency distributions of the difference in the estimated dS of the subhit and of the best hit divided by the sum of their standard error. The distributions are significantly different indicating that dS is generally lower when the ortholog in the ancestral position.

We then considered only the comparisons of the most closely related homologs, where both best hits and sub hitshad estimated dS of less than 3, therefore more reliable. A total of 365 such gene pairs were considered in this analysis (see methods). Again, we considered cases where the BBH was not the positional homolog but a duplicate homolog. The difference in the dS for the subhit and the dS for the BBH was estimated. The frequency distribution of these values is plotted in Figure 4. As expected, we observed significantly different dS values (p=4.2×10^−6^) when the BBH were positional homologs in comparison to when the homologs were in a distinct genome positions. There was however, no significant difference in the dN values of those two groups (not shown, p=0.17). These results did not change when smaller subsets of gene pairs with still lower dS values were considered (data not shown). Our results indicate that DNA sequence divergence is reduced for positional homologs compared to when the homologs (of comparable protein sequence divergence) are in a different genomic position.

The lowered dS estimates of the positional homologs is additional evidence that gene order conservation is indicative of the orthology of the genes since the sequences are, on average, less diverged at the DNA level compared to other copies even though they are equally, or more diverged, at the protein level.

### Best strategy for finding orthologs from only homologous hits

**Figure 5:**
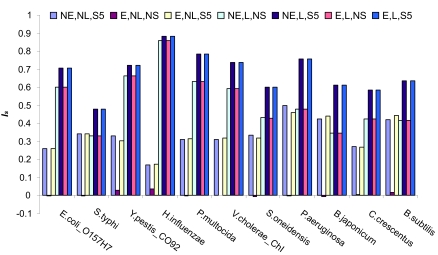
Positional homolog identification score of several cut-off strategies in selected *E. coli* K12 comparisons The Positional homolog identification score *I_s_* is given for different BLAST cut off strategies in several comparisons with the *E. coli* genome. NE: Large E-value cut-off (E < 10) NL: Alignment length > 30 amino acids. NS: No subhit cut-off E: E-value cut-off (E < 0.01) L: Length cut-off (70% of larger protein) S5: No subhits within 10^5^× E-value.

Simple Blast analyses are often performed for pairwise genome comparisons to indicate homologous hits. Having determined the number of BBH that could be confirmed as positional or duplicate homologs based on gene neighbor information, we then assessed different strategies for identifying positional homologs when additional genomes, gene order and DNA sequence were not considered. We wanted to find the combination of BLAST (Altschul 1997) and FASTA (Pearson 1994) parameters that identified the largest numbers of positional homologs and minimized the numbers of duplicate homologs found in pairwise genome comparisons. For such comparisons, the addition of several requirements for maximum E-value, E-value spread and minimum percentage alignment length were considered. E-value cutoffs and sequence coverage cut-offs are routinely used for BBH screening. The positional homolog identification score (*I_s_,* from equation 1) was determined in each case for all genome comparisons. A graph of the scores for selected comparisons with *E. coli* K12 is given in Figure 5. Table 2 displays the average score over all the genome comparisons. The results show that imposing a requirement of a minimum E-value of 0.01 on the BBH did not result in a significant improvement in the scores. We did not increase the stringency further since even this low level resulted in reduced numbers of identified potential positional homologs (and lower scores).

As expected, adding a minimum length requirement greatly improved the accuracy of prediction, with the more stringent requirement of 70% coverage over the longer protein performing better than the more lenient requirement of 70% coverage of the shorter protein. This analysis also showed a difference in performance between BLAST and FASTA. A minimum length requirement is more important for BLAST than for FASTA since even the Gapped-BLAST (the version of BLAST used) returned shorter, more local alignments, and it was more prone to the identification of paralogous domains than was FASTA. Once a minimum alignment length was applied however, BLAST analysis gave higher average *I_s_* scores.

**Figure 6:**
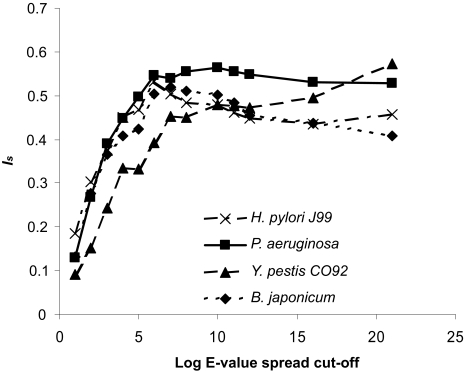
Positional homolog identification score versus E-value spread The behavior of the Positional homolog identificaton score *I_s_* with increasing E-value spread (but no other stringency requirements) is shown for a few genome comparisons for BBH identified by BLAST. The E-value spread on the X-axis is given as the log base 10 of the multiplier for the E-value within which no subhits are allowed. The E-value spread for which *I_s_* is maximum varies from comparison to comparison.

The largest score improvement was obtained by putting a strict E-value spread requirement. E-value spread is defined as the ratio of the best hit and the next best hit E-values. This last requirement does not place a threshold on the E-value of the BBH itself, but does set a threshold on any subhits (as a function of the BBH E-value). Theoretically, the score should increase with stringency up to a maximum, slowly decrease, and eventually level off with an extremely high spread requirement. This plateau will be reached only when queries with single hits and no significant subhits are considered. The level of E-value spread required for obtaining the maximum, and thus optimal positional homolog identification score, depends on the pair of genomes under consideration. There may even be several local maxima. Thus, it is difficult to determine a uniform standard that should be applied for E-value spreads. In general, the level should be higher for comparisons of more closely related genomes than for more divergent comparisons. This is shown in Figure 6 where the positional homolog identification score *I_s_*, is plotted with an increasing stringency of E-value spread, for a sample of genome comparisons.

The success of the E-value spread approach for identifying positional homologs makes sense. If there are no other close homologs in the genome, then the chance of having identified a duplicate copy is low, whereas if there is evidence of other homologs in the genome, then we cannot be certain of the orthology of the BBH. An efficient way of correctly identifying positional homologs is then to require BBH to have a strong alignment coverage requirement and a strict E-value spread. A BBH that does not meet these criteria should be considered suspect because even though it may still indicate an ortholog, there is evidence for another ortholog in what can be considered the ancestral position, such that the BBH may be an unreliable maker for genome phylogeny based on gene order.

Interestingly, the positional homolog identification score was not necessarily highest for the evolutionarily closest comparisons. This is because, although the number of duplicates was low for these comparisons, the BBH identified were very good hits that can be difficult to eliminate with even stringent E-value cut-offs. Increasing the stringency of alignment coverage from 70% of the shortest protein to 70% of the longest protein did remove some of the duplicate identifications in the evolutionarily close comparison of *E. coli* strains, indicating that length conservation could also be used as an indicator of orthology in these comparisons.

The *I_s_* score as defined in Eq. 1, ignores the unconfirmed hits. These indicate genes for which the gene order is not conserved but for which there is no evidence of another positional homolog. As an indicator of orthology, not just positional homology, a less conservative *I_s_* score can be determined by including the unconfirmed hits as positive. The *I_s_* score can also be made a more stringent measure of positional homology by including the unconfirmed BBH as negative hits. We also investigated these alternative measures and found similar results (data not shown). Our analysis, using positional homology as an indicator of accuracy for the identification of single orthologs in pairwise genome comparisons demonstrates that accuracy is increased with larger E-value spread between best hits and subhits.

### Positional homologs in COGs

The COG database is an approach for identifying orthologs (Tatusov *et al.* 1997, Tatusov *et al.* 2000, Tatusov *et al.* 2001) that does not consider E-value and coverage cutoffs for BBH between pairs of genomes, but instead uses the hit information of a third genome to confirm orthology. This is a better way of identifying orthologs than simply considering the BBH, but requires more information than the two genomes under comparison. When there has been lineage-specific expansion of a gene family within a genome, paralogs are grouped into the same COG cluster. These paralogs have been called “co-orthologs” or “in-paralogs” by Sonnhammer and Koonin (2002), and define multiple orthologous pairs when compared to homologous sequences in other genomes. When present, gene order conservation can help to identify the most ancestral orthologous copy for one gene from the group of duplicated genes in another genome. Many of the entries in the COG database are BBH that can also be confirmed with gene order information, but it is interesting to consider cases where a secondary hit is identified as the positional homolog. We considered the subset of the 3505 total duplicates identified in our 123-genome comparisons with BLAST in the 66 genomes of the COG database. From those 1772 duplicates, we found 1210 instances (68%) where these gene-pairs were included and assigned to the same COG. This indicates that the database does contain a significant number of in-paralogous identifications for which gene order is instructive. A sample of these in comparisons involving the *E. coli* K12 genome is shown in Table 3 and the complete set from all genome comparisons is available as supplementary materials (Supplementary Table). The tables give the duplicate BBH and also the sub-hit corresponding to the positionaly conserved homolog. Not surprisingly, many of the duplicates are members of large gene families (transporters and chaperonins, for example).

## Discussion

We used a strategy that attempts to identify the positional homology of genes and help confirm the orthology of proteins using gene order information. The premise is that if two genes are BBH and their neighboring genes are also BBH, it is therefore most likely that the pairs are orthologs. However lack of positional homology does not indicate paralogy, as gene order may have been lost, or duplications may have occurred after speciation of the two genomes considered. In the case of a lineage specific gene family expansion, gene order is still informative however, as it can indicate the original copy of the gene from which duplicates arose, providing additional information. This strategy may also identify homologs where functional constraints, such as co-regulation of gene expression in operons, select for multiple recombination events (Jordan *et al*., 2001) or when lateral gene transfer and replacement of the ancestral sequence (Omelchenko *et al*. 2003) has occurred. These scenarios are not so unlikely since we have shown that there is more DNA sequence homology at positional homologs than at other duplicate locations, allowing for homologous recombination. Lateral gene transfer and recombination replacement has even been shown to occur within a single gene (Omenchelko *et al*., 2003), such that the evolutionary lineage of any particular gene is difficult to ascertain in bacteria. For the study of gene order evolution, it is however still reasonable to make the parsimonious argument that genes in the same order should be considered ancestrally orthologous.

When segmental duplications occurred, whereby groups of contiguous genes are duplicated, then using the strict neighbors of a gene cannot be used to ascertain positional homology since these have also been duplicated. In the current analysis we first screened for segmental duplications by finding contiguous hits within a genome and flagged such pairs. Hits from to these genes in other genomes were then labeled Ambiguous. This is conservative because one of the two neighbors is often not part of the segmental duplication and could, therefore be used to determine ancestry. When neighbors are part of the segmental duplication event, theoretically, the ambiguity could be resolved if we considered neighboring genes further away until we identify ones that are not part of the segmental duplication. Implementation of such strategies requires additional assumptions about gene conservation and their order within segmental duplications among different genomes.

Most often, BBH identify positional homologs, but there are also cases where the positional homolog is not the most conserved at the protein sequence level and will thus not be identified as the best alignment. The number is highest when genomes are at an intermediate level of divergence, i.e., when there is still some gene order conservation but with reduced sequence identities (Huynen and Bork 1998). We found that in many cases, the DNA sequence alignment is better for the positional homologs than for the duplicate. When chance, recombination or positive selection leads to increased protein sequence divergence of genes in the ancestral locations, then there is a greater chance of BBH identifying duplicate homologs and not the positional homologs.

Using the property of gene order conservation in related genomes, we could separate the identified BBH between genomes in ancestral positions from duplicate copies in different positions. We then determined the best strategy to remove the duplicate identifications without consideration of gene order. The analysis gives an indication of the best approach to use for finding single orthologous pairs when the gene order is unknown or is not conserved between the genomes of interest. We found that the first main requirement is to use a minimum length for the hits, such as covering 70% of the larger gene. As it has been shown previously, this eliminates hits due to shared domains and is particularly important for BLAST analyses. The second necessary criterion necessary is to apply a stringent E-value filter for subsequent hits since these can indicate dubious ortholog identification or the presence of co-orthologs. With this strategy, the actual E-value threshold for BBH can be very low and the number of identified BBH maximized. Diverged sequences with low similarity can be included, but only when there is no evidence of duplication.

Gene order information provides additional information that can, in some cases, be used to identify ancestral positional orthologs where sequence alone does not permit us to differentiate the ancestral copy from a multitude of duplicates. We have shown the limits of this approach, since gene order information is not informative in cases of segmental duplications and is therefore not as applicable to eukaryotic genomes. Gene order conservation has also provided us with a useful gauge for optimizing BLAST search parameters which is extremely important because pairwise hits often form the basis of comparative genomic studies that consider multiple genomes (Remm *et al*. 2001, Uchiyama 2003). It can also be used in conjunction with other ortholog finding methods such as COG and INPARANOID (Tatusov *et al*. 2001, O’Brien *et al*. 2005) to identify the positional, and thus, most likely ancestral orthologs. We have found that additional information from the DNA sequence could also be used to differentiate the homologs since we show that in many cases the DNA sequence in the ancestral location would reveal a better alignment than the duplicate. The results for accurate assignment of the positional homologs will be most useful when accuracy of the historical relationships of gene copies is vital such as when building genome phylogenies based on gene order and in studies of the co-evolution of genes and their regulatory elements.

## Figures and Tables

**Table 1:** Groups of segmental duplications for selected bacterial genomes

Genome	Groups	Genes	Genome percentage	Average # genes per group
*Escherichia coli* O157H7	98	804	15.05	8.20
*Streptomyces coelicolor*	191	1175	14.41	6.15
*Bradyrhizobium japonicum*	173	1108	13.32	6.40
*Pseudomonas aeruginosa*	113	598	10.74	5.29
*Yersinia pestis* CO92	62	415	10.20	6.69
*Escherichia coli* K12	78	425	9.86	5.45
*Mycobacterium tuberculosis* CDC1551	70	388	9.27	5.54
*Salmonella typhi*	68	403	8.47	5.93
*Bacillus halodurans*	63	330	8.12	5.24
*Bacillus subtilis*	57	307	7.47	5.39
*Shewanella oneidensis*	50	303	6.78	6.06
*Vibrio cholerae*	54	256	6.68	4.74
*Pirellula sp.*	80	468	6.39	5.85
*Streptococcus mutans*	20	82	4.18	4.10
*Caulobacter crescentus*	32	143	3.83	4.47
*Pasteurella multocida*	15	75	3.72	5.00
*Haemophilus influenzae*	10	51	3.08	5.10
*Mycobacterium leprae*	11	47	2.93	4.27
*Helicobacter pylori* J99	9	34	2.28	3.78

**Table 2:** Mean positional homolog identification scores (Is) using BLAST and FASTA with various E-value cutoffs, E-value spreads and minimum length cut-offs

	E-value Cut-off	Length Cut-off	None	E-value Spread 100 × E-value	10^5^ × E-value
**Blast**	E≤10	No Length	0.00^a^ (0.00)^b^	0.228 (0.141)	0.332 (0.159)
	E≤0.01	No Length	0.021 (0.040)	0.222 (0.138)	0.328 (0.158)
	E≤10	70% Shorter Protein	0.469 (0.210)	0.632 (0.158)	0.632 (0.158)
	E≤0.01	70% Shorter Protein	0.419 (0.211)	0.565 (0.167)	0.648 (0.157)
	E≤10	70% Longer Protein	0.613 (0.179)	0.692 (0.144)	**0.746** (0.136)
	E≤0.01	70% Longer Protein	0.612 (0.179)	0.691 (0.145)	0.745 (0.138)

**Fasta**	E≤10	No Length	0.00 (0.00)	0.293 (0.134)	0.390 (0.157)
	E≤0.01	No Length	0.059 (0.083)	0.286 (0.130)	0.385 (0.157)
	E≤10	70% Shorter Protein	0.380 (0.200)	0.559 (0.170)	0.618 (0.262)
	E≤0.01	70% Shorter Protein	0.380 (0.201)	0.555 (0.171)	0.620 (0.264)
	E≤10	70% Longer Protein	0.566 (0.195)	0.699 (0.156)	**0.758** (0.131)
	E≤0.01	70% Longer Protein	0.564 (0.196)	0.697 (0.158)	0.755 (0.133)

a *I_s_* from 123 pairwise genome comparisons,

b deviation

**Table 3:** Selected duplicate and positional homologs from *E. coli* K12 in the COG database.

*E.coli* K12 PID	Species Y	Species Y PID BBH (Duplicate)	COG #	COG Name	SubHit PID (Positional)	SubHit Evalue
16128318	*B.halodurans*	15615722	COG0372	Citrate synthase Alpha-galactosidases/6-phospho-beta-	15616486	4.00E-66
16129688	*B.halodurans*	15612746	COG1486	glucosidases, family 4 of glycosyl hydrolases	15613475	1.00E-109
16131449	*B.halodurans*	15615234	COG1593	Integral membrane protein, possible transporter	15613266	1.00E-69
16129639	*B.subtilis*	16080319	COG0719	Predicted membrane components of an uncharacterized iron-regulated ABC-type transporter SufB	16080322	1.00E-39
16129445	*B.japonicum*	27376148	COG0601	ABC-type dipeptide/oligopeptide/nickel transport systems, permease components	27378449	4.00E-64
16130014	*B.japonicum*	27379567	COG0845	Membrane-fusion protein	27375387	1.00E-69
16131325	*B.japonicum*	27376144	COG1653	Sugar-binding periplasmic proteins/domains	27375844	1.00E-116
16129701	*C.crescentus*	16125853	COG3138	Arginine/ornithine N-succinyltransferase beta subunit	16124835	8.00E-51
16131968	*C.glutamicum*	19553910	COG0459	Chaperonin GroEL (HSP60 family)	19551832	1.00E-157
16130275	*M.tuberculosis* CDC1551	15839624	COG0183	Acetyl-CoA acetyltransferases	15840272	3.00E-39
16132208	*P.aeruginosa*	15599791	COG0488	ATPase components of ABC transporters with duplicated ATPase domains	15598215	4.00E-94
16128299	*P.aeruginosa*	15599128	COG01292	Choline-glycine betaine transporter	15600568	1.00E-131
16129086	*V.cholerae*	15641435	COG0687	Spermidine/putrescine-binding periplasmic protein	15641436	1.00E-113
16130181	*Y.pestis* CO92	16121833	COG0477	Permeases of the major facilitator superfamily	16122022	3.00E-63

## List of abbreviations

BBH: Bi-directional best hits
ORF: Open reading frame
